# Supplementary material for: Predation and fragmentation portrayed in the statistical structure of prey time series
Source: BMC Ecol. 2009 May 6;9:10. doi: 10.1186/1472-6785-9-10 (PMC2689204; doi:10.1186/1472-6785-9-10)
Supplement: Additional file 2 — Voles and related classes ODDox Documentation. ODDox documentation of the agent-based model (ALMaSS) applied by Hendrichsen et al. The documentation is started by activating main.html. [file 1472-6785-9-10-S2.zip › Vole_ODDox/globals_0x74.html]

ALMaSS ODDox: Class Members

- Main Page
- Related Pages
- Classes
- Files

- File List
- File Members

- All
- Functions
- Variables
- Typedefs
- Enumerations
- Enumerator
- Defines

- \_
- a
- b
- c
- d
- f
- g
- h
- i
- l
- m
- n
- o
- p
- r
- s
- t
- u
- v
- w

Here is a list of all file members with links to the files they belong to:

### - t -

- TheGestationPeriod
  : Vole\_all.cpp- TListOfAnimals
    : PopulationManager.h- TListOfPredators
      : Predators.H- tof\_AgroChemIndustryCerealFarm1
        : farm.h- tof\_AgroChemIndustryCerealFarm2
          : farm.h- tof\_AgroChemIndustryCerealFarm3
            : farm.h- tof\_ConventionalCattle
              : farm.h- tof\_ConventionalPig
                : farm.h- tof\_ConventionalPlant
                  : farm.h- tof\_ConvMarginalJord
                    : farm.h- tof\_NoPesticideBase
                      : farm.h- tof\_NoPesticideNoP
                        : farm.h- tof\_OrganicCattle
                          : farm.h- tof\_OrganicPig
                            : farm.h- tof\_OrganicPlant
                              : farm.h- tof\_PTrialControl
                                : farm.h- tof\_PTrialToxicControl
                                  : farm.h- tof\_PTrialTreatment
                                    : farm.h- tof\_UserDefinedFarm1
                                      : farm.h- tof\_UserDefinedFarm10
                                        : farm.h- tof\_UserDefinedFarm11
                                          : farm.h- tof\_UserDefinedFarm12
                                            : farm.h- tof\_UserDefinedFarm13
                                              : farm.h- tof\_UserDefinedFarm14
                                                : farm.h- tof\_UserDefinedFarm15
                                                  : farm.h- tof\_UserDefinedFarm16
                                                    : farm.h- tof\_UserDefinedFarm2
                                                      : farm.h- tof\_UserDefinedFarm3
                                                        : farm.h- tof\_UserDefinedFarm4
                                                          : farm.h- tof\_UserDefinedFarm5
                                                            : farm.h- tof\_UserDefinedFarm6
                                                              : farm.h- tof\_UserDefinedFarm7
                                                                : farm.h- tof\_UserDefinedFarm8
                                                                  : farm.h- tof\_UserDefinedFarm9
                                                                    : farm.h- tole\_ActivePit
                                                                      : tole\_declaration.h- tole\_BeetleBank
                                                                        : tole\_declaration.h- tole\_Building
                                                                          : tole\_declaration.h- tole\_Coast
                                                                            : tole\_declaration.h- tole\_ConiferousForest
                                                                              : tole\_declaration.h- tole\_DeciduousForest
                                                                                : tole\_declaration.h- tole\_Field
                                                                                  : tole\_declaration.h- tole\_FieldBoundary
                                                                                    : tole\_declaration.h- tole\_Foobar
                                                                                      : tole\_declaration.h- tole\_Freshwater
                                                                                        : tole\_declaration.h- tole\_Garden
                                                                                          : tole\_declaration.h- tole\_Heath
                                                                                            : tole\_declaration.h- tole\_HedgeBank
                                                                                              : tole\_declaration.h- tole\_Hedges
                                                                                                : tole\_declaration.h- tole\_LargeRoad
                                                                                                  : tole\_declaration.h- tole\_Marsh
                                                                                                    : tole\_declaration.h- tole\_MixedForest
                                                                                                      : tole\_declaration.h- tole\_NaturalGrass
                                                                                                        : tole\_declaration.h- tole\_Orchard
                                                                                                          : tole\_declaration.h- tole\_PermanentSetaside
                                                                                                            : tole\_declaration.h- tole\_PermPasture
                                                                                                              : tole\_declaration.h- tole\_PermPastureLowGrazing
                                                                                                                : tole\_declaration.h- tole\_PitDisused
                                                                                                                  : tole\_declaration.h- tole\_Railway
                                                                                                                    : tole\_declaration.h- tole\_River
                                                                                                                      : tole\_declaration.h- tole\_RiversidePlants
                                                                                                                        : tole\_declaration.h- tole\_RiversideTrees
                                                                                                                          : tole\_declaration.h- tole\_RoadsideVerge
                                                                                                                            : tole\_declaration.h- tole\_Saltwater
                                                                                                                              : tole\_declaration.h- tole\_Scrub
                                                                                                                                : tole\_declaration.h- tole\_SmallRoad
                                                                                                                                  : tole\_declaration.h- tole\_StoneWall
                                                                                                                                    : tole\_declaration.h- tole\_Track
                                                                                                                                      : tole\_declaration.h- tole\_UnsprayedFieldMargin
                                                                                                                                        : tole\_declaration.h- tole\_Urban
                                                                                                                                          : tole\_declaration.h- tole\_YoungForest
                                                                                                                                            : tole\_declaration.h- tops\_Dispersal
                                                                                                                                              : Predators.H- tops\_Hunting
                                                                                                                                                : Predators.H- tops\_InitialState
                                                                                                                                                  : Predators.H- tops\_Movement
                                                                                                                                                    : Predators.H- tov\_AgroChemIndustryCereal
                                                                                                                                                      : tov\_declaration.h- tov\_Carrots
                                                                                                                                                        : tov\_declaration.h- tov\_CloverGrassGrazed1
                                                                                                                                                          : tov\_declaration.h- tov\_CloverGrassGrazed2
                                                                                                                                                            : tov\_declaration.h- tov\_FieldPeas
                                                                                                                                                              : tov\_declaration.h- tov\_FieldPeasStrigling
                                                                                                                                                                : tov\_declaration.h- tov\_FodderBeet
                                                                                                                                                                  : tov\_declaration.h- tov\_Maize
                                                                                                                                                                    : tov\_declaration.h- tov\_MaizeStrigling
                                                                                                                                                                      : tov\_declaration.h- tov\_NaturalGrass
                                                                                                                                                                        : tov\_declaration.h- tov\_NoGrowth
                                                                                                                                                                          : tov\_declaration.h- tov\_None
                                                                                                                                                                            : tov\_declaration.h- tov\_Oats
                                                                                                                                                                              : tov\_declaration.h- tov\_OBarleyPeaCloverGrass
                                                                                                                                                                                : tov\_declaration.h- tov\_OCarrots
                                                                                                                                                                                  : tov\_declaration.h- tov\_OCloverGrassGrazed1
                                                                                                                                                                                    : tov\_declaration.h- tov\_OCloverGrassGrazed2
                                                                                                                                                                                      : tov\_declaration.h- tov\_OCloverGrassSilage1
                                                                                                                                                                                        : tov\_declaration.h- tov\_OFieldPeas
                                                                                                                                                                                          : tov\_declaration.h- tov\_OFieldPeasSilage
                                                                                                                                                                                            : tov\_declaration.h- tov\_OFirstYearDanger
                                                                                                                                                                                              : tov\_declaration.h- tov\_OGrazingPigs
                                                                                                                                                                                                : tov\_declaration.h- tov\_OOats
                                                                                                                                                                                                  : tov\_declaration.h- tov\_OPermanentGrassGrazed
                                                                                                                                                                                                    : tov\_declaration.h- tov\_OPotatoes
                                                                                                                                                                                                      : tov\_declaration.h- tov\_Orchard
                                                                                                                                                                                                        : tov\_declaration.h- tov\_OSBarleySilage
                                                                                                                                                                                                          : tov\_declaration.h- tov\_OSeedGrass1
                                                                                                                                                                                                            : tov\_declaration.h- tov\_OSeedGrass2
                                                                                                                                                                                                              : tov\_declaration.h- tov\_OSetaside
                                                                                                                                                                                                                : tov\_declaration.h- tov\_OSpringBarley
                                                                                                                                                                                                                  : tov\_declaration.h- tov\_OSpringBarleyClover
                                                                                                                                                                                                                    : tov\_declaration.h- tov\_OSpringBarleyGrass
                                                                                                                                                                                                                      : tov\_declaration.h- tov\_OSpringBarleyPigs
                                                                                                                                                                                                                        : tov\_declaration.h- tov\_OTriticale
                                                                                                                                                                                                                          : tov\_declaration.h- tov\_OWinterBarley
                                                                                                                                                                                                                            : tov\_declaration.h- tov\_OWinterRape
                                                                                                                                                                                                                              : tov\_declaration.h- tov\_OWinterRye
                                                                                                                                                                                                                                : tov\_declaration.h- tov\_OWinterWheatUndersown
                                                                                                                                                                                                                                  : tov\_declaration.h- tov\_PermanentGrassGrazed
                                                                                                                                                                                                                                    : tov\_declaration.h- tov\_PermanentGrassLowGrazed
                                                                                                                                                                                                                                      : tov\_declaration.h- tov\_PermanentSetaside
                                                                                                                                                                                                                                        : tov\_declaration.h- tov\_Potatoes
                                                                                                                                                                                                                                          : tov\_declaration.h- tov\_PotatoesIndustry
                                                                                                                                                                                                                                            : tov\_declaration.h- tov\_SeedGrass1
                                                                                                                                                                                                                                              : tov\_declaration.h- tov\_SeedGrass2
                                                                                                                                                                                                                                                : tov\_declaration.h- tov\_Setaside
                                                                                                                                                                                                                                                  : tov\_declaration.h- tov\_SpringBarley
                                                                                                                                                                                                                                                    : tov\_declaration.h- tov\_SpringBarleyCloverGrass
                                                                                                                                                                                                                                                      : tov\_declaration.h- tov\_SpringBarleyCloverGrassStrigling
                                                                                                                                                                                                                                                        : tov\_declaration.h- tov\_SpringBarleyGrass
                                                                                                                                                                                                                                                          : tov\_declaration.h- tov\_SpringBarleyPeaCloverGrassStrigling
                                                                                                                                                                                                                                                            : tov\_declaration.h- tov\_SpringBarleySeed
                                                                                                                                                                                                                                                              : tov\_declaration.h- tov\_SpringBarleySilage
                                                                                                                                                                                                                                                                : tov\_declaration.h- tov\_SpringBarleyStrigling
                                                                                                                                                                                                                                                                  : tov\_declaration.h- tov\_SpringBarleyStriglingCulm
                                                                                                                                                                                                                                                                    : tov\_declaration.h- tov\_SpringBarleyStriglingSingle
                                                                                                                                                                                                                                                                      : tov\_declaration.h- tov\_SpringRape
                                                                                                                                                                                                                                                                        : tov\_declaration.h- tov\_SpringWheat
                                                                                                                                                                                                                                                                          : tov\_declaration.h- tov\_Triticale
                                                                                                                                                                                                                                                                            : tov\_declaration.h- tov\_Undefined
                                                                                                                                                                                                                                                                              : tov\_declaration.h- tov\_WinterBarley
                                                                                                                                                                                                                                                                                : tov\_declaration.h- tov\_WinterBarleyStrigling
                                                                                                                                                                                                                                                                                  : tov\_declaration.h- tov\_WinterRape
                                                                                                                                                                                                                                                                                    : tov\_declaration.h- tov\_WinterRapeStrigling
                                                                                                                                                                                                                                                                                      : tov\_declaration.h- tov\_WinterRye
                                                                                                                                                                                                                                                                                        : tov\_declaration.h- tov\_WinterRyeStrigling
                                                                                                                                                                                                                                                                                          : tov\_declaration.h- tov\_WinterWheat
                                                                                                                                                                                                                                                                                            : tov\_declaration.h- tov\_WinterWheatShort
                                                                                                                                                                                                                                                                                              : tov\_declaration.h- tov\_WinterWheatStrigling
                                                                                                                                                                                                                                                                                                : tov\_declaration.h- tov\_WinterWheatStriglingCulm
                                                                                                                                                                                                                                                                                                  : tov\_declaration.h- tov\_WinterWheatStriglingSingle
                                                                                                                                                                                                                                                                                                    : tov\_declaration.h- tov\_WWheatPControl
                                                                                                                                                                                                                                                                                                      : tov\_declaration.h- tov\_WWheatPToxicControl
                                                                                                                                                                                                                                                                                                        : tov\_declaration.h- tov\_WWheatPTreatment
                                                                                                                                                                                                                                                                                                          : tov\_declaration.h- tov\_YoungForest
                                                                                                                                                                                                                                                                                                            : tov\_declaration.h- tovm\_Infanticide
                                                                                                                                                                                                                                                                                                              : VolePopulationManager.H- tovs\_FDying
                                                                                                                                                                                                                                                                                                                : vole\_all.h- tovs\_FEvaluateExplore
                                                                                                                                                                                                                                                                                                                  : vole\_all.h- tovs\_FMaturation
                                                                                                                                                                                                                                                                                                                    : vole\_all.h- tovs\_GiveBirth
                                                                                                                                                                                                                                                                                                                      : vole\_all.h- tovs\_Infanticide
                                                                                                                                                                                                                                                                                                                        : vole\_all.h- tovs\_InitialState
                                                                                                                                                                                                                                                                                                                          : vole\_all.h- tovs\_JuvenileExploration
                                                                                                                                                                                                                                                                                                                            : vole\_all.h- tovs\_Lactating
                                                                                                                                                                                                                                                                                                                              : vole\_all.h- tovs\_Mating
                                                                                                                                                                                                                                                                                                                                : vole\_all.h- tovs\_MDying
                                                                                                                                                                                                                                                                                                                                  : vole\_all.h- tovs\_MEvaluateExplore
                                                                                                                                                                                                                                                                                                                                    : vole\_all.h- tovs\_MMaturation
                                                                                                                                                                                                                                                                                                                                      : vole\_all.h- tovs\_ReproBehaviour
                                                                                                                                                                                                                                                                                                                                        : vole\_all.h- tovs\_SpecialExplore
                                                                                                                                                                                                                                                                                                                                          : vole\_all.h- tovs\_UpdateGestation
                                                                                                                                                                                                                                                                                                                                            : vole\_all.h- trial\_control
                                                                                                                                                                                                                                                                                                                                              : treatment.h- trial\_insecticidetreat
                                                                                                                                                                                                                                                                                                                                                : treatment.h- trial\_toxiccontrol
                                                                                                                                                                                                                                                                                                                                                  : treatment.h- TTypeOfPredatorState
                                                                                                                                                                                                                                                                                                                                                    : Predators.H- TTypeOfVoleMessage
                                                                                                                                                                                                                                                                                                                                                      : VolePopulationManager.H- TTypeOfVoleState
                                                                                                                                                                                                                                                                                                                                                        : vole\_all.h- TTypesOfFarm
                                                                                                                                                                                                                                                                                                                                                          : farm.h- TTypesOfLandscapeElement
                                                                                                                                                                                                                                                                                                                                                            : tole\_declaration.h- TTypesOfVegetation
                                                                                                                                                                                                                                                                                                                                                              : tov\_declaration.h

---

Generated on Thu Jan 22 14:13:48 2009 for ALMaSS ODDox by 
 1.5.6
